# Vitamin D Receptor Signaling and Ligand Modulation: Molecular Mechanisms and Therapeutic Implications

**DOI:** 10.3390/ijms27052396

**Published:** 2026-03-04

**Authors:** Tram Thi-Ngoc Nguyen, Kouki Nojiri, Tomohiro Kurokawa, Takahiro Sawada, Yoshiaki Kanemoto, Shigeaki Kato

**Affiliations:** 1 Department of Pharmacy, Iryo Sosei University, Chuodai Iino, Iwaki 970-8551, Fukushima, Japan; 2 Research Institute of Innovative Medicine, Tokiwa Foundation, Iwaki 971-8112, Fukushima, Japan; 3 School of Medicine, Fukushima Medical University, Fukushima 960-1295, Fukushima, Japan

**Keywords:** vitamin D receptor (VDR), synthetic analogs, selective VDR modulators, hypercalcemia

## Abstract

Vitamin D, a fat-soluble vitamin functioning as a hormone via the vitamin D receptor (VDR), is critical for calcium homeostasis and bone health. Vitamin D deficiency is linked to nutritional rickets, osteomalacia, and increased risk of non-communicable diseases such as cancer and diabetes. While serum 25(OH)D_3_ is used to assess vitamin D status, its active form, 1α,25(OH)_2_D_3_, exerts context-dependent effects on calcium metabolism. Nonetheless, the therapeutic utility of native vitamin D is limited in certain pathologies. In chronic kidney disease (CKD), the renal conversion of 25(OH)D_3_ to active 1α,25(OH)_2_D_3_ is compromised, necessitating the use of active synthetic analogs to bypass this metabolic defect. Furthermore, for dermatological and oncological disorders requiring supraphysiological dosing, synthetic analogs have been designed to dissociate beneficial anti-proliferative effects from the severe hypercalcemia induced by high-dose 1α,25(OH)_2_D_3_. VDR mediates transcriptional responses, modulated by co-regulators and chromatin remodeling complexes. Recent discoveries include non-genomic VDR pathways and SCAP (SREBP cleavage-activating protein)-dependent signaling that modulate lipid metabolism. Despite promising preclinical results, most synthetic VDR agonists fail to show efficacy in cancer therapy due to calcemic toxicity. However, compounds like eldecalcitol are effective in osteoporosis, especially in low-calcium-intake populations. Selective VDR modulators, akin to SERMs, exhibit tissue-specific effects. Moreover, novel VDR antagonists such as ZK168281 demonstrate potential to suppress hypercalcemia and vitamin D toxicity by inhibiting transcriptional activity and altering VDR localization. These agents may enable anti-inflammatory or anti-proliferative actions without calcemic risks. Understanding the nuanced biology of vitamin D and its analogs offers new avenues for therapeutic intervention beyond bone metabolism, including managing hyperparathyroidism, granulomatous diseases, and inflammation-associated disorders.

## 1. Introduction

**Figure 1:**
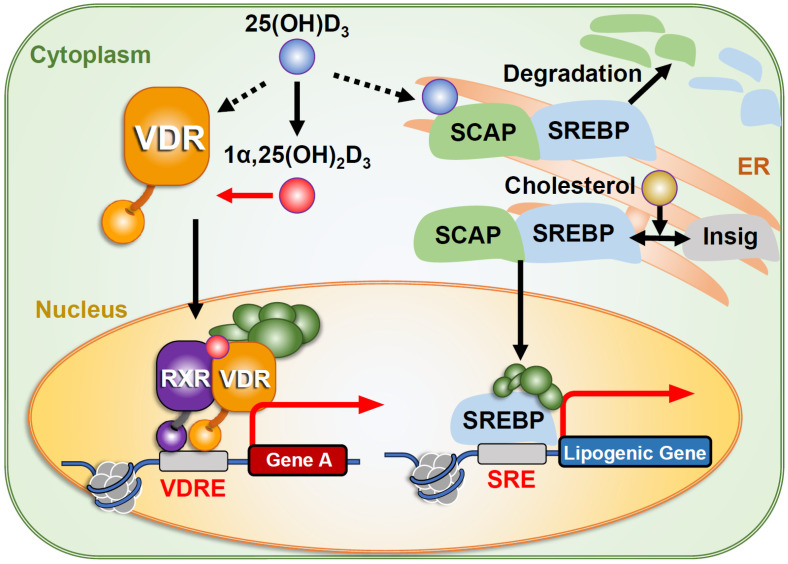
**Intracellular Signaling Pathways of Vitamin D**. The active form of vitamin D, 1,25(OH)_2_D_3_, serves as the most potent ligand for the nuclear vitamin D receptor (VDR) [12]. It is generated via hydroxylation of its precursor, 25(OH)D_3_. In addition to binding VDR, 25(OH)D_3_ can also bind to SREBP cleavage-activating protein (SCAP), inhibiting SREBP-mediated activation of lipogenic genes [13].

Vitamin D is a fat-soluble vitamin that also functions as a hormone, acting as a natural ligand for the nuclear vitamin D receptor (VDR) (Figure 1) [1]. Vitamin D plays diverse biological roles and is most well-known for supporting bone growth and maintenance by regulating calcium homeostasis [2,3,4]. Children who are deficient in vitamin D, calcium, and/or phosphorus or who have genetic defects may develop nutritional rickets or hypophosphatemic rickets. As calcium and phosphate metabolisms are tightly connected, the defects in phosphate metabolism-related genes such as PHEX (phosphate-regulating endopeptidase homolog, X-linked) and FGF (fibroblast growth factor) 23 can also cause hypophosphatemic rickets [5,6]. This review discusses genes related to VDR-mediated vitamin D signaling (Figure 1). Nutritional rickets is characterized by inadequate mineralization of epiphyseal plates, impaired bone growth, bony deformities, and soft bones. Adults with nutritional rickets also develop softening of the bones or osteomalacia [2], which occurs following epiphyseal plate fusion. To prevent or reduce the likelihood of these conditions, the therapeutic use of cod liver oil and exposure to sunlight was recommended approximately a century ago [7]. In subsequent decades, nutritional forms of vitamin D were isolated and synthetic derivatives were developed [8]. Researchers later identified the forms of vitamin D in serum and elucidated the mechanisms regulating its concentrations in vivo. Since then, the physiological functions of vitamin D have been studied extensively [9], and its molecular actions have been clarified through the development of vitamin D-related compounds [10].

More recently, epidemiological studies have shown a significant association between vitamin D deficiency and the incidence of certain cancers [9,11], but the association remains mechanistically elusive. Although clinical trials have tested synthetic vitamin D analogs as anti-cancer agents, they have not demonstrated substantial efficacy in either preventing or regressing various tumor types [9]. This lack of clinical efficacy may be due in part to dose limitations, as higher doses of vitamin D increase the likelihood of inherent calcemic effects [9,10].

## 2. Literature Search and Selection Strategy

This article is a narrative review focusing on the molecular mechanisms and therapeutic development of synthetic vitamin D receptor (VDR) ligands. The literature was identified through comprehensive searches of PubMed, Web of Science, and Google Scholar. using combinations of keywords including “vitamin D receptor,” “synthetic vitamin D analogs,” “selective VDR modulators,” “hypercalcemia,” “ligand-binding domain,” and “coregulator recruitment.” Additional references were obtained by manual screening of the reference lists of key review articles and seminal original studies.

Studies were selected based on their relevance to VDR biology, ligand structure–function relationships, transcriptional mechanisms, and clinical or preclinical evaluation of synthetic vitamin D analogs. Priority was given to peer-reviewed articles providing mechanistic insight, structural information, or representative clinical evidence, rather than exhaustive coverage of all available reports. As a narrative review, no formal inclusion or exclusion criteria or quantitative synthesis was applied; instead, the cited literature reflects the authors’ expert assessment of studies most informative for understanding the evolution, mechanisms, and therapeutic prospects of VDR-targeting compounds.

## 3. Nutritional Status of Vitamin D and Disease Incidence

The most biologically active form of vitamin D is 1α,25(OH)_2_D_3_, which has the highest affinity for VDRs. However, its concentration in serum is low (16–50 pg/mL) and it is rarely used as a marker of vitamin D status, as its local concentrations among target tissues and cells are different and do not reflect its serum level. Instead, its precursor, 25(OH)D_3_, is widely used for biochemical assessments. Serum concentrations of 25(OH)D_3_ usually range from 10 to 70 ng/mL, with concentrations above 50 ng/mL being considered sufficient. Vitamin D below the 20 ng/mL level indicates insufficiency, and levels below 10 ng/mL indicate a moderate to severe deficiency [2,14,15,16].

Adults over 70 years old are particularly susceptible to vitamin D insufficiency, primarily due to reduced dietary intake, decreased intestinal absorption, and decreased sun exposure [17]. Large-scale clinical surveys from multiple countries have shown that most individuals, particularly the elderly, do not maintain sufficient vitamin D concentrations [15,16]. Notably, significant associations have been reported between vitamin D insufficiency and the incidence of non-communicable diseases such as cancer, hypertension, and diabetes [18]. Although the molecular mechanisms linking vitamin D status to these diseases remain unclear, impaired immune function is believed to be a contributing factor [19,20]. For example, vitamin D may enhance the activation of monocytes and macrophages, as well as the induction of antimicrobial peptides like cathelicidins, reflecting its role in immune responses [21,22,23]. As anti-proliferative action of vitamin D has been also documented in many human cell lines [24,25], nutritional deficiency of vitamin D may promote incidence and development of cancer [26].

## 4. Calcemic Action of Vitamin D and Bone Health

Vitamin D is one of the key calcemic hormones, along with parathyroid hormone (PTH) and fibroblast growth factor 23 (FGF23), and it acts on calcium-regulating organs such as the intestine, kidneys, and bones [27]. These hormones cooperate to maintain calcium homeostasis, with each playing distinct roles in regulating systemic calcium levels. Under conditions of positive calcium balance, vitamin D promotes calcium deposition in bone by enhancing intestinal calcium absorption and renal calcium reabsorption, while parathyroid hormone (PTH) levels are suppressed, thereby limiting bone resorption. Conversely, during a negative calcium balance, vitamin D acts in concert with elevated PTH to increase bone resorption and renal calcium conservation to maintain serum calcium levels. In parallel, fibroblast growth factor 23 (FGF23), produced by osteocytes, modulates this response by suppressing renal phosphate reabsorption and inhibiting excess vitamin D activation, thereby preventing hyperphosphatemia and aberrant mineralization; sustained FGF23 signaling also contributes to the suppression of bone matrix mineralization under conditions of mineral imbalance [28,29].

Therefore, under negative calcium balance, bone mass is further decreased in osteoporotic patients. Calcium balance is mainly dependent on the dietary intake, but pathological conditions such as hypophosphatemia and hyperparathyroidism modulate calcium balance, leading adverse action of vitamin D in bone metabolism [5,30]. Thus, vitamin D exerts context-dependent effects on bone metabolism, depending on the body’s calcium status. While it is essential for bone health, excessive vitamin D activity can lead to hypercalcemia, regardless of calcium status [28,31].

## 5. Regulation of Serum Levels of Vitamin D

**Figure 2:**
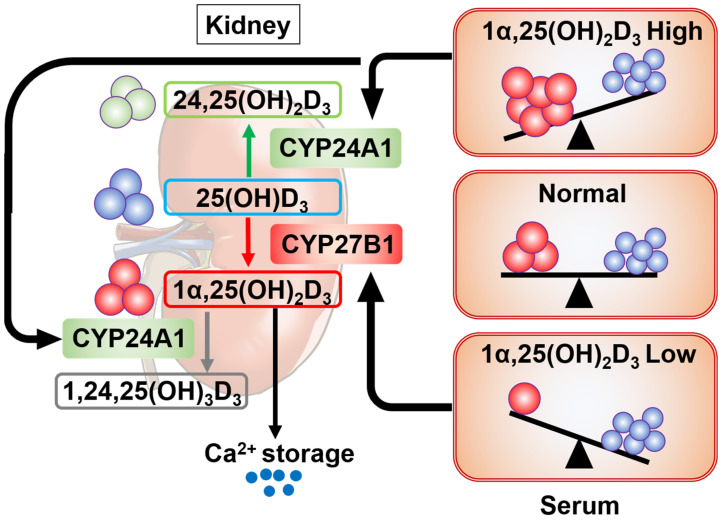
**Biosynthesis and Regulation of 1,25(OH)_2_D_3_**. Serum concentrations of 1,25(OH)_2_D_3_ are tightly controlled by two key enzymes [33]. In the kidney, CYP27B1 catalyzes the conversion of 25(OH)D_3_ into the active form, 1,25(OH)_2_D_3_. This active form is subsequently inactivated by CYP24A1, which converts it to 24,25(OH)_2_D_3_. These reactions maintain vitamin D homeostasis in response to physiological demands.

To meet the body’s calcium demands, serum 1α,25(OH)_2_D_3_ is tightly regulated by multiple mechanisms (Figure 2). A key step in the biosynthesis of vitamin D is the conversion of 25(OH)D_3_ into 1α,25(OH)_2_D_3_ via 1α-hydroxylation by the renal enzyme CYP27B1 [32,33]. This enzymatic activity is positively regulated by parathyroid hormone (PTH) and negatively regulated by 1α,25(OH)_2_D_3_ at the level of transcription [32,34]. When 1α,25(OH)_2_D_3_ concentrations become excessive, 1α,25(OH)_2_D_3_ is inactivated by conversion into 1,24,25(OH)_2_D_3_ through 24-hydroxylation, catalyzed by CYP24A1 in the kidney and other tissues [32]. Notably, *CYP24A1* is a highly inducible target gene of VDR, and its expression is robustly upregulated by ligand-bound VDR [35,36]. In addition to known protein transcription factors, such as the retinoid X receptor (RXR), which activates the *CYP24A1* promoter, non-coding RNAs appear to facilitate gene induction by vitamin D through chromatin reorganization. Specifically, several types of non-coding RNAs (ncRNAs) have been identified as VDR targets. For instance, a long non-coding RNA (lncRNA) is transcribed from the antisense strand of the human *HSD17B2* locus in response to vitamin D. Furthermore, enhancer RNAs (eRNAs) act as epigenetic factors to reorganize the chromatin environment, facilitating gene regulation by the VDR complex [35,37].

Beyond renal metabolism, a significant aspect of vitamin D biology involves its extrarenal activation. While the kidney maintains systemic calcium homeostasis through tightly regulated 1α,25(OH)_2_D_3_ (Figure 2), various extrarenal cells—most notably, macrophages and monocytes—also express CYP27B1 [38,39]. Unlike the renal enzyme, extrarenal CYP27B1 is not primarily regulated by PTH or calcium but is instead induced by inflammatory stimuli such as toll-like receptor (TLR) signaling [23,38]. This local conversion allows 1α,25(OH)_2_D_3_ to act in an autocrine or paracrine fashion to modulate immune responses, including the induction of antimicrobial peptides like cathelicidins [40]. Because this extrarenal production is largely substrate-dependent, maintaining sufficient serum 1α,25(OH)_2_D_3_ concentrations is critical for effective immunomodulation, independent of systemic calcium demands.

## 6. Key Factors in Vitamin D Signaling—Lessons from Hereditary Rickets

**Figure 3:**
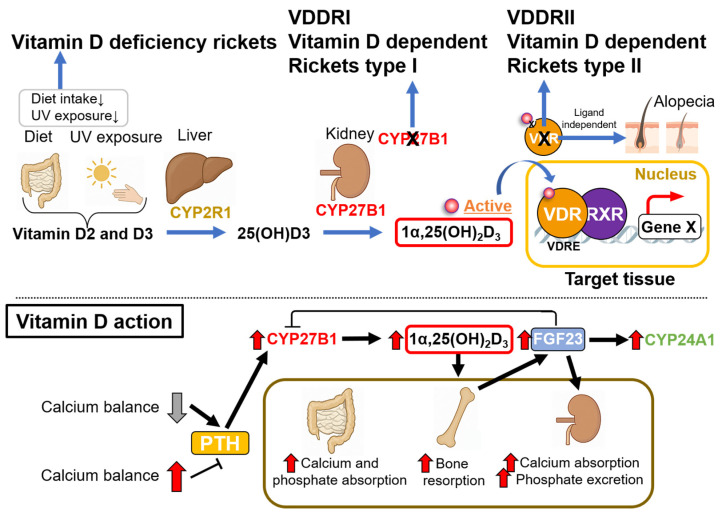
Schematic and brief view of vitamin D action in whole body and rickets by nutritious deficiency and defects of vitamin D signaling.

Hereditary rickets is categorized into two distinct types based on specific genetic defects. Vitamin D-dependent rickets type I (VDDRI) is caused by inactivating mutations in the *CYP27B1* gene, which encodes the 1α-hydroxylase enzyme required to convert 25(OH)D_3_ into the active form 1α,25(OH)_2_D_3_ [41]. In contrast, vitamin D-dependent rickets type II (VDDRII) arises from null or malfunctioning mutations in the VDR gene (Figure 3) [42]. Despite these distinct etiologies, both forms exhibit abnormalities similar to those seen in subjects with nutritional vitamin D deficiency (Figure 3) [4,33,43]. Typical rachitic defects in the growing stage include growth attenuation due to bone growth retardation, while osteomalacia emerges in adults with nutritional deficiency. Both bone growth retardation and malformation result from lowered levels of serum calcium, as vitamin D is the prime calcemic hormone in calcium homeostasis by stimulating intestinal calcium absorption and renal calcium reabsorption. Thus, hypocalcemia is commonly seen in all types of rickets subjects, and severe hypocalcemia further induces hyperparathyroidism and hyperphosphatemia, leading to more severe bone defects. Clinically, type II rickets is distinguishable by the appearance of alopecia, which serves as a diagnostic marker [42], while Type I rickets patients are indistinguishable in terms of overt abnormalities from subjects with nutritional vitamin D deficiency [44]. Experimentally, these two responsible genes were verified as key factors in vitamin D signaling by rachitic abnormalities in mouse lines with genetically disrupted genes (VDR and CYP27B1 KO mice) [45,46,47]. Molecular dissection of the two rickets mouse models has provided insight into the physiological function of VDR in intact animals.

Nutritional vitamin deficiency or genetically malfunction of CYP27B1 and VDR can cause the onset of rickets during growth. In rachitic adults, osteomalacia emerges [2,3,4,9]. Precursors of vitamin D (vitamin D2 and vitamin D3) acquired from the diet or biosynthesized on skin are converted into the active form of vitamin D, 1,25(OH)_2_D_3_, by hepatic CYP2R1 and renal CYP27B1. When levels of 1,25(OH)_2_D_3_ in the body are excessive, 1,25(OH)_2_D_3_ and 25(OH)D_3_ in serum are catabolized into inactive forms of the vitamin. Among the natural forms of vitamin D, 1,25(OH)_2_D_3_ exhibits the highest affinity as a VDR ligand, and activated VDR regulates positive and negative expression of a particular set of target genes in a tissue-specific manner [48,49,50]. Hereditary type I rickets involves malfunction of CYP27B1 and type II includes malfunction of VDR [51]. Mechanistically, the VDR function in hair formation is ligand-independent; thus, VDR mutants lacking ligand binding activity are often sufficient for normal hair formation without alopecia in type II patients [52,53]. Clinically, null function of VDR in type II patients is associated with alopecia as a diagnosis marker, which is not observed in rachitic patients with nutritional deficiency and malfunction of CYP27A1 [51].

## 7. Function of VDR in Vitamin D Signaling

### 7.1. Activated VDR Function by Ligand Binding Is Indispensable for Bone Growth

Hypocalcemia in type I rickets patients as well as in *CYP27B1* gene knock-out (KO) mice is ameliorated by simply providing 1α(OH)D_3_ or 1α,25(OH)_2_D_3_ [41,46]. Tissue-specific VDR gene disruption by a Cre-loxP approach has demonstrated the physiological significance of the VDR function of vitamin D actions in mouse intestinal tissues [54]. Ligand-dependent VDR function in regulation of the VDR target genes has been confirmed by transcriptome analyses of KO mouse tissues and gene expression profiles in cultured cells. Replacing amino acid residues in the LBD domains of mouse and human VDRs has successfully enabled mapping of amino acid residues that are essential for ligand binding and subsequent transactivation function [12,55,56]. Consistently, mutations in VDR genes found in rickets patients were mostly found in the coding regions for core functional domains of DNA binding (such as cysteine 60 to tryptophan and arginine 73 to glutamine mutations) and ligand binding, in the VDR protein of 427 amino acid residues. However, serious rachitic defects in mineral and bone metabolism accommodate genetic mutations (such as arginine 343 to cysteine and serine 360 to proline) in the C-terminal end of VDR, which is necessary for ligand-dependent association with transcriptional co-regulators [37,51]. Ligand-dependency of VDR function in serum calcium regulation has been more intensively studied in rat lines with introduced mutations (arginine 270 to leucine and histidine 301 to glutamine) in the VDR LBD identified in type II patients [57]. Administration of 1α,25(OH)_2_D_3_ is not effective to ameliorate hypocalcemia in animals with mutated VDR genes and in type II patients. However, calcium supplementation to address hypocalcemia in such rickets patients and animals is effective enough to rescue growth retardation and osteomalacia by normalizing bone formation [58], implying that vitamin D action on bone development during growth is mainly indirect through positive serum calcium regulation acting on calcium handling tissues such as the intestine and kidney. Under negative calcium balance, VDR activated by vitamin D transcriptionally induces osteoclastogenic genes in osteoblasts to induce bone resorption and calcium release from bone. Under negative calcium balance, VDR activated by vitamin D transcriptionally induces osteo-clastogenic genes in osteoblasts to induce bone resorption for calcium release from bone. In contrast, VDR induces genes in osteoblasts to facilitate bone formation with attenuating expression of osteoclastogenic genes under positive calcium balance [59,60]. As bone growth retardation emerges only after weaning in the VDR KO mice and the rickets pa-tients [43,45], the physiological significance of vitamin D-VDR signaling in calcium homeostasis is evident only after intake not of milk but of a post-weaning diet that does not contain vitamin D in meaningful concentrations. These observations establish that ligand-dependent regulation of the vitamin D target genes by VDR underlies the calcemic action of vitamin D to support bone growth and remodeling.

### 7.2. Unliganded VDR Is Essential for Skin Maturation

Alopecia is visible only in type II rickets patients with null function of VDR, that are mutated or deficient in function of DNA binding, nuclear translocation, and/or co-regulator interaction [45], since DNA binding for VDR requires a heterodimeric partner, one of the RXR subtypes (RXRα, RXRβ, and RXRγ) [37,61]. However, mutations resulting only in lack of ligand binding are not associated with risk of alopecia [55]. Lessons from genetically manipulated rodent lines confirmed that VDR mutants defective of ligand binding were able to support normal hair growth without hair loss [52,53,62], indicating that unliganded VDR is sufficient for normal hair development. More detailed analysis of the hair follicles of VDR mutants showed that presence of VDR is essential for hair follicle homeostasis [63]. Lack of VDR was shown to halt the hair formation cycle at the catagen phase in the regenerating hair follicles, leading to loss of hair [45,53]. Similar alopecia was also seen in mice lacking a transcriptional co-regulator (Hairless), which further proved to serve as a co-repressor for VDR in cultured skin cells [64]. These observations further imply that unliganded VDR associating transcriptional co-repressors such as Hairless suppresses expression of the target genes in skin. This idea is further supported by in vitro observations that unliganded VDR bound to DNA acts as transcriptionally suppressive for transcription by associating with co-repressors [65]. Thus, VDR is considered to be critical for normal hair cycle maintenance by suppressing target genes in a specific type of skin cells, and ligand binding appears dispensable for VDR function in skin [43,56].

### 7.3. Indirect and Direct Action of Vitamin D Mediated by Vitamin D on Bone

Vitamin D is essential for bone health in humans, but its action on bone remodeling appears complicated by a close link with calcium homeostasis. In intact animals and humans, vitamin D is beneficial for bone metabolism and health, as vitamin D supplementation under normal calcium balance in the whole body is prone to deposit calcium into bone [27]. The pivotal function of VDR in calcium homeostasis controlled by vitamin D has been verified experimentally with the use of VDR KO mice and rats [45,52], and clinically in human type II rickets patients [42,43]. Severe bone loss with hypocalcemia was shown in adult VDR KO mice with hyperparathyroidism, which enhances bone resorption [37]. However, similar to type II rickets patients, calcium supplementation to VDR KO mice was sufficiently effective to recover hypocalcemia and osteomalacia and also restore growth retardation [45,58]. These observations in humans and rodents establish a view that vitamin D action on bone growth and remodeling is mainly indirectly mediated by serum calcium under normal or positive calcium balance. Locally sufficient concentrations of calcium with phosphate facilitate bone formation by mineralization with the aid of bone matrix proteins such as collagen type I, osteocalcin, osteopontin, and dentin matrix protein 1 (DMP1), leading to increased bone mass [27,28]. For bone cells, vitamin D can be assumed to directly act to finely tune bone growth and remodeling, under various calcium conditions within the whole body.

Historically, the direct action of vitamin D on bone was assessed using organ cultures of bone or co-culture of osteoblast and osteoclasts [66,67]. Regarding those results together with recent findings, the in vitro action of vitamin D is generally considered to stimulate bone resorption as a stimulator for osteoclastogenesis, inducing the gene expression of osteoclastogenic factor, RANKL (receptor activator of NFκB ligand) in osteoblasts [68]. In contrast, its osteoblastogenic action in in vitro culture settings looks marginal. To address this inconsistent view of skeletal vitamin D action on bone mass control between in vivo and vitro, conditional VDR KO to ablate VDR in calcium handling tissues was introduced, and careful experiments using these mouse lines have been conducted under various nutritional calcium statuses [54,69]. VDR genes have been ablated in osteoblasts at different stages of cell differentiation by Cre expression under control of stage-specific active gene promoters (Osterix for osteoprogenitors, Col1a1 for immature osteoblasts) [69,70]. Under normal dietary conditions, ablation of VDR by Cre expression driven by the Osterix promoter is assumed to be associated with defective vitamin D action in the osteogenic promoter. As these mice basically exhibited no abnormality in bone mass and bone metabolism, it is likely that VDR function activated by vitamin D is marginal for osteogenesis [69]. A modest increase in bone mass with decreased bone turnover was seen in mice with ablated VDR in immature osetoblasts using Cre driven by the *Col1a* promoter [64]. From the observations from this line, it appears that osteoblastic VDR is suppressive for bone resorption. These mouse lines exhibited certain but not robust bone phenotypes, basically suggesting that VDR is dispensable for osteoblastgenesis and following cell differentiation. Interestingly, bone resorption appears more affected by VDR ablation in osteoblastic cells than bone formation [63], and these observations are supportive for those of in vitro cultured organs reported previously [66,67]. It appears from the gene expression analysis of these conditional VDR KO mice lines that activated VDR by vitamin D in osteoblastic cells in the whole body regulates expression of the osteoclastic regulator, RANKL, consistent with in vitro observations [59,69]. Thus, osteoblastic VDR appears significant for bone resorption and the subsequent coupled regulation of bone turnover. [59,69].

### 7.4. Vitamin D-Dependent Function of VDR in Calcium Handling Tissues for Calcium Homeostasis

Intestinal ablation of VDR in mice (int-VDR KO) was shown to result in hypocalcemia as well as bone mass decrease due to low intestinal absorption of calcium from the intestine, similar to VDR KO mice [54]. When VDR was overexpressed driven by the villin prompter in int-VDR KO mice, more remarkable expression of VDR in the intestine was detected than in wild type mice. However, under negative calcium conditions induced by feeding a low-calcium diet, decrease in bone mass was not attenuated [71]. Thus, for vitamin D-controlled normal balance of calcium in the whole body, intestinal VDR is important but not sufficient, requiring the VDR function in the other calcium-handling tissues. The significance of VDR in vitamin D-dependent suppression of calcium excretion in the kidneys has been proven through high urinary calcium excretion in systemic KO mice lines of VDR as well as CYP27B1 [46]; it is essential for converting into the active form of vitamin D. For calcium transport in renal cells, TRPV5/6, Calbindin (CaBP)-28K, and CaBP-9K are identified as pivotal factors, and expressions of TRPV5, CaBP-28K, and CaBP-9K are under the control of VDR-mediated vitamin D action [72]. Since ablation of each gene of these three vitamin D-dependent factors did not fully restore the phenotypes in terms of calcium homeostasis and bone defects, all of the three factors appear required to maintain VDR-mediated vitamin D action for renal function associated with calcium homeostasis in the whole body [27]. However, this does not exclude the possibility of the presence of unknown factors other than these three factors, as the ablation of all of the three genes in mice remains to be tested. Thus, vitamin D-dependent VDR plays a central role in calcium homeostasis in calcium-handling tissues, but it is notable that calcium homeostasis is also controlled by the other calcemic hormones, PTH, and FGF23, and the VDR function is modulated by the actions of these hormones.

## 8. Signaling Pathway of Vitamin D

Most of the biological actions of vitamin D are mediated through the VDR (Figure 1). The nuclear form of VDR acts as a transcription factor [4,45,73], whereas the membrane-associated VDR may trigger rapid, non-genomic responses, although the physiological relevance of these non-genomic pathways remains uncertain [74,75]. As vitamin D induces cellular events rapidly (within seconds or a few minutes), several cellular factors binding or/and associating vitamin D are considered to constitute VDR-independent/non-genomic pathways. For instance, several kinases were shown to be activated rapidly by vitamin D treatments in cultured cells, but the physiological significance of such candidate factors in the biological activity of vitamin D remains to be elusive in whole animals [74,76]. More recently, another molecule that directly binds to 25(OH)D_3_ has been identified, SREBP cleavage-activating protein (SCAP), a sterol-binding protein (Figure 1). SCAP retains SREBPs in the endoplasmic reticulum (ER) membrane by forming a complex with insulin-induced genes (INSIGs), which serve as ER anchor proteins [13]. When 25(OH)D_3_ binds to SCAP instead of sterols, SCAP undergoes self-ubiquitination and is degraded, leading to SREBP degradation [13,77]. Although this mechanism has been demonstrated in cell lines, its physiological role in vivo remains to be clarified. Nevertheless, it may partially explain how vitamin D suppresses lipid synthesis.

## 9. Gene-Regulatory Functions of VDR

VDR is a member of the nuclear receptor (NR) superfamily and is encoded by a single gene locus in higher vertebrates [4,73,78]. Similar to thyroid hormone receptors (TRs) and retinoic acid receptors (RARs), VDR forms heterodimers with one of the three retinoid X receptor isoforms (RXRα, RXRβ, or RXRγ), which function as ligand-independent partners. The typical DNA binding site for the VDR/RXR heterodimer consists of two 5′-HGGTCA-3′ motifs separated by a three-base spacer [79]. Upon ligand binding, the VDR/RXR complex binds to vitamin D response elements (VDREs), modulating gene transcription [80,81]

VDR is a ligand-dependent transcription factor that regulates gene expression in a cell type-specific manner. Although many protein-coding genes have been identified as transcriptional targets of vitamin D/VDR, they alone cannot account for the full spectrum of biological functions of vitamin D. More recently, several types of non-coding RNAs (ncRNAs), including long non-coding RNAs and enhancer RNAs (eRNAs), have been identified as VDR targets [35,73,82,83]. As eRNA acts as epigenetic factor to reorganize chromatin environment among ncRNAs, vitamin D-dependent eRNAs may facilitate gene regulations by vitamin D [37,84]. While the biological functions of most VDR-regulated ncRNAs remain unclear, they may contribute to both known and yet-undiscovered actions of vitamin D [2,3,4,78].

## 10. Transcriptional Co-Regulators Facilitating VDR Function

Unlike many classical steroid hormone receptors, such as the glucocorticoid, androgen, and progesterone receptors, which are predominantly retained in the cytosol in association with heat-shock proteins and translocate to the nucleus upon ligand binding, the vitamin D receptor (VDR) is localized in both the cytosol and nucleus in most target cells. VDR functions as a member of the nuclear receptor superfamily and forms heterodimers with the retinoid X receptor (RXR), binding to vitamin D response elements (VDREs) in the promoter or enhancer regions of target genes. Although VDR is primarily nuclear in the absence of ligand, binding of 1,25-dihydroxyvitamin D_3_ enhances its nuclear accumulation, stabilizes VDR–RXR complexes on chromatin, and promotes the recruitment of coactivator complexes, thereby increasing transcriptional activity [80,81].

**Figure 4:**
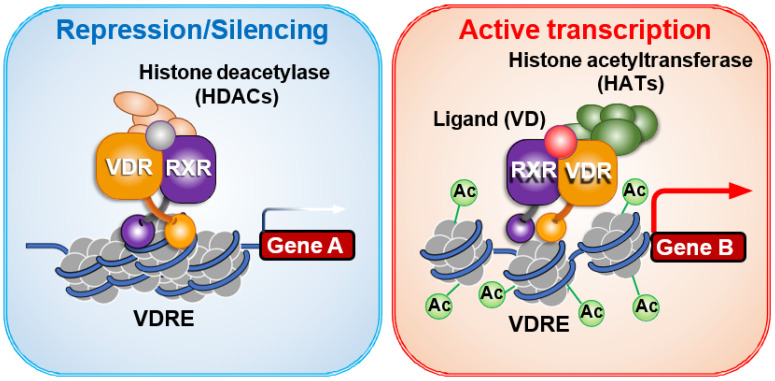
**Ligand-Dependent Switching of VDR Co-regulators**. In the absence of ligand, the vitamin D receptor (VDR) remains transcriptionally inactive, even when bound to VDR elements, by recruiting co-repressors such as histone deacetylases [65]. Upon ligand binding, VDR undergoes a conformational change that leads to the dissociation of co-repressors and recruitment of co-activators like histone acetyltransferases, facilitating chromatin relaxation and transcriptional activation [37].

To exert its transcriptional activity, ligand-bound VDR requires transcriptional co-regulators. These co-regulators assemble with the mediator complex to initiate transcription via RNA polymerase II [48,49,50]. In the absence of a ligand, VDR is transcriptionally inactive and associates with co-repressors such as histone deacetylases (HDACs) [48,49,50,85]. Ligand binding induces the release of these co-repressors and recruitment of co-activators (Figure 4), accompanied by a conformational change in the C-terminal region of VDR.

Co-regulators can be broadly classified into three groups [48,49,78]. The first includes general co-activators like the mediator complex, which connects DNA-bound transcription factors (such as NRs) with the general transcription machinery [86]. The second group consists of histone-modifying enzymes, including HDACs (co-repressors) and histone acetyltransferases, that function as co-activators in a ligand-dependent manner [48,87]. Although histone methylation/demethylation enzymes are also important for transcription [88], their involvement in VDR-mediated regulation appears limited [89,90].

The third group comprises chromatin remodeling complexes that modify local chromatin structure to facilitate transcription [88], and a role has been reported for chromatin remodeling complexes in VDR function in pathological progression in pancretatic β- cells [87]. However, studies using VDR-deficient human cells have shown that the global landscape of chromatin assessed by histone modifications and chromatin openness was not significantly changed in mutated cells treated with or without vitamin D [89]. From these observations, it is unlikely that VDR is able to remodel chromatin by means of chromatin remodeling complexes.

Numerous synthetic vitamin D derivatives have been developed based on the chemical structure of biologically active vitamin D compounds [10]. Several of these analogs have demonstrated promising effects in experimental settings. As a result, synthetic vitamin D ligands have been evaluated in clinical trials for their potential to prevent cancer or suppress tumor progression. However, none have shown sufficient clinical efficacy [10]. In contrast, eldecalcitol, a synthetic VDR ligand, has been clinically effective as an anti-osteoporotic drug [91,92]. The clinical effectiveness of eldecalcitol is mostly observed in countries such as Japan, where low dietary calcium intake may enhance the drug’s benefits, particularly in elderly individuals with reduced intestinal calcium absorption [37]. In cell culture models, various synthetic analogs exhibit different biological activities, depending on their chemical structure, which affects their affinity for VDR and their transcriptional potency [10]. These compounds may also act through alternative pathways, such as those involving SCAP or potentially via unidentified membrane receptors [13,74]. However, it is most likely that VDR is the target of synthetic vitamin D analogues.

## 11. Historical Development of Synthetic VDR Ligands

### 11.1. Early Recognition of VDR and Calcitriol Analogs

The VDR was first identified in the 1970s as the nuclear receptor responsible for the genomic effects of calcitriol. By the late 1970s and early 1980s, the receptor’s ability to regulate calcium and bone metabolism had become well established. However, therapeutic use of calcitriol is severely constrained by hypercalcemia, particularly in conditions requiring high or chronic dosing, such as chronic kidney disease or cancer.

This limitation prompted medicinal chemists to begin synthesizing structural analogs of calcitriol. The earliest efforts focused on subtle modifications of the secosteroid backbone of vitamin D, altering side-chain length, hydroxylation patterns, or double bonds. These efforts laid the groundwork for developing analogs with greater potency in transcriptional regulation but reduced calcemic activity [93,94].

### 11.2. Expansion of Analog Design in the 1980s–1990s

By the late 1980s, analog development accelerated, producing the first clinically tested synthetic ligands. Compounds such as EB1089 (seocalcitol) emerged as potent anti-proliferative agents with reduced hypercalcemic risk in animal models [95,96]. Around the same time, calcipotriol (MC903) was introduced for dermatological use, providing an effective topical treatment for psoriasis [97,98]. As both have less affinity for serum vitamin D binding protein (DBP), their rapid clearance in serum leads to less calcemic action.

In the 1990s, parallel advances in molecular biology provided tools to study how synthetic ligands altered VDR transcriptional activity. It became clear that certain modifications not only reduced hypercalcemia but also biased VDR toward specific co-regulator recruitment, giving rise to the idea of selective VDR modulators (SVDRMs) [99].

### 11.3. Clinical Translation and Diversification

By the early 2000s, several analogs reached clinical application. Paricalcitol (19-nor-1,25D_3_) was approved for secondary hyperparathyroidism in chronic kidney disease [93,100], while maxacalcitol (OCT) entered use in Japan for psoriasis [101]. The field expanded from dermatology and nephrology to oncology and immunology, where synthetic ligands showed promise in modulating proliferation, apoptosis, and immune tolerance [102].

More recently, non-secosteroidal ligands have been explored, representing a departure from traditional vitamin D backbone design. These compounds aim to overcome pharmacokinetic limitations, offering improved stability, tissue selectivity, and oral bioavailability (See Table 1) [103,104].

**Table 1:** Comparison of Key Synthetic Vitamin D Analogues and VDR Modulators.

Analogue	Chemical Modification	Pathology	Key Mechanism	Outcome/Limitation
**EB1089** (Seocalcitol)	Side-chain modification [105]	Cancer (hepatocellular, pancreatic, breast) [106]	Induces apoptosis via p53-independent p38 MAPK activation; stabilizes antiproliferative VDR conformation [102]	**Limitation:** Mixed clinical outcomes due to pharmacokinetic challenges.
**Calcipotriol** (MC903)	Side-chain modification [99]	Psoriasis [107]	Selective antiproliferative effect on keratinocytes; rapid serum clearance reduces systemic calcemic risk [99]	**Outcome:** Highly successful topical treatment, often combined with corticosteroids [108].
**Paricalcitol** (19-nor-1,25D_3_)	“19-nor” modification (removal of carbon 19) [93]	Secondary hyperparathyroidism (CKD) [93,100,109]	Suppresses PTH levels with reduced calcemic activity compared to calcitriol [93]	**Limitation:** Therapeutic window remains narrow due to inherent calcemic and phosphatemic activity [110].
**Maxacalcitol** (OCT)	Side-chain modification [111]	Psoriasis [101]	Selectively targets skin keratinocytes with limited systemic calcemic action [101]	**Outcome:** Clinically approved and effective in Japan [101].
**Eldecalcitol**	Alterations in the A-ring [112]	Osteoporosis [91,92]	Potent inhibition of bone resorption; structure-based design for tissue selectivity [91]	**Outcome:** Effective in increasing bone mineral density, particularly in populations with low dietary calcium [91,92].
**ZK168281**	Side-chain modification [99]	Hypercalcemia; Vitamin D toxicity [113]	Inhibits transcriptional activity; retains VDR in the cytosol by interacting with WBP4 [114,115]	**Outcome:** Reverses hypercalcemia and gene upregulation in vivo; potential for treating hypervitaminosis D [116].
**Non-secosteroidal Ligands** (e.g., LG190178)	Non-secosteroidal backbone (lacks classic steroid rings)	Various (experimental)	Improved metabolic stability and tissue selectivity; mimics key hydrogen bonding of VDR [103,117]	**Limitation:** Currently in preclinical development; not yet widely used clinically [118,119,120,121].

## 12. Structural Insights into Synthetic VDR Ligand Binding

### 12.1. The Ligand-Binding Domain of VDR

The ligand-binding domain (LBD) of VDR consists of approximately 12 α-helices forming a typical nuclear receptor fold. Central to ligand function is helix 12 (H12), also known as the activation function-2 (AF-2) helix, which undergoes conformational changes upon ligand binding [122,123,124]. These conformational shifts determine whether the receptor preferentially recruits coactivators or corepressors, and thus whether transcription is activated or repressed [125,126,127]. Synthetic ligands interact with the hydrophobic binding pocket of the VDR, stabilizing distinct conformations of H12. Even minor modifications in ligand structure can shift the equilibrium toward agonism, partial agonism, or antagonism [128,129].

### 12.2. Insights from Crystallography and SAR Studies

The first crystallographic structures of VDR bound to calcitriol and synthetic ligands, solved in the late 1990s and early 2000s, provided a foundation for structure–activity relationship (SAR) studies [99,125]. These studies revealed that the following:

- Modifications at the side chain (C20–C25 region) significantly influence calcemic activity [99];
- Alterations in the A-ring and triene system affect receptor binding affinity and transcriptional potency [99];
- Non-secosteroidal scaffolds, though structurally distinct, can mimic key hydrogen bonding patterns critical for stabilizing the ligand–receptor complex [103].

For example, EB1089 was found to stabilize VDR in a conformation that promoted antiproliferative effects while producing less calcemic response [95]. Calcipotriol, in contrast, modified the side chain to enhance selectivity for keratinocytes, explaining its success as a topical agent [99].

### 12.3. Selective Modulation and Tissue Targeting

One of the most important insights from structural studies is that different ligands induce distinct conformational states, leading to selective transcriptional outcomes. This supports the concept of ligand bias, where synthetic compounds can direct VDR toward gene- or tissue-specific regulation [128]. In practice, this allows design of ligands that are effective in skin or parathyroid tissue without significantly affecting intestinal calcium absorption [130].

## 13. Noteworthy Synthetic VDR Ligands

### 13.1. EB1089 (Seocalcitol)

Developed in the late 1980s, EB1089 (seocalcitol) represents a pioneering attempt to dissociate the potent antiproliferative effects of vitamin D from its calcemic toxicity [92]. Structurally, EB1089 features a modified side chain containing a terminal ethyl group, which alters its interaction with the VDR ligand-binding domain compared to the natural ligand. Mechanistically, EB1089 has been shown to induce apoptosis in cancer cells (e.g., hepatocellular and pancreatic carcinoma) through unique signaling cascades. Unlike classical genomic VDR signaling, it triggers the p53-independent activation of p38 mitogen-activated protein kinase (MAPK) and suppresses ERK activity [99], as well as downregulating the anti-apoptotic protein Bcl-2 [128]. Despite promising preclinical data, clinical translation was hindered. Phase II/III trials in hepatocellular carcinoma and pancreatic cancer revealed that while EB1089 was less calcemic than calcitriol, outcomes were mixed due to pharmacokinetic challenges [129] and dose-limiting hypercalcemia that prevented achieving the high concentrations required for tumor regression. Although development was discontinued, EB1089 remains an invaluable benchmark compound for investigating the molecular basis of VDR-mediated anticancer effects [130].

### 13.2. Calcipotriol (MC903)

Calcipotriol (MC903), introduced in the early 1990s, established the paradigm for topical vitamin D therapy [106]. Its design specifically addressed the need for a potent VDR agonist that could be applied to skin without inducing systemic hypercalcemia. Structurally, it incorporates a cyclopropane ring in the side chain and a hydroxyl group at position 24. The clinical success of calcipotriol relies on its unique pharmacokinetic profile. Calcipotriol exhibits a significantly lower affinity for the serum vitamin D binding protein (DBP) compared to 1,25(OH)_2_D_3_ [96]. This low affinity renders it susceptible to rapid enzymatic degradation in the liver once it enters the systemic circulation [131]. Consequently, while it potently activates VDR in skin keratinocytes, any drug absorbed into the blood is cleared too quickly to impact intestinal calcium absorption. It remains a first-line treatment for psoriasis vulgaris [107], often used in combination with corticosteroids.

### 13.3. Paricalcitol (19-nor-1,25D_3_)

Paricalcitol (19-nor-1,25-dihydroxyvitamin D2) is approved for the treatment of secondary hyperparathyroidism in chronic kidney disease (CKD) [90,97,108]. Its structural modification involves the removal of the exocyclic carbon-19 atom and the inclusion of a vitamin D2 side chain, which confers “tissue selectivity”. Unlike calcitriol, paricalcitol induces a specific VDR conformation that favors the recruitment of co-regulators essential for suppressing parathyroid hormone (PTH) gene transcription, while recruiting intestinal co-activators less efficiently [109]. This differential action provides a wider therapeutic window, suppressing PTH with a reduced risk of hypercalcemia compared to calcitriol, although the therapeutic window is narrow [110]. Its widespread clinical use underscores the success of rational design in achieving tissue-selective VDR modulation [132].

### 13.4. Maxacalcitol (OCT)

Maxacalcitol (22-oxacalcitriol), approved in Japan for psoriasis, exemplifies regional differences in clinical translation [98]. Structurally characterized by the substitution of the carbon atom at position 22 with an oxygen atom (22-oxa), this modification dramatically reduces its binding affinity for DBP [98]. Similar to calcipotriol, the low DBP affinity allows maxacalcitol to exist primarily in a free form for rapid tissue uptake and clearance. In Japan, maxacalcitol has achieved a unique clinical status: it is approved not only as a topical ointment for psoriasis but also as an intravenous injection for secondary hyperparathyroidism [117]. It effectively suppresses PTH secretion with a manageable calcemic profile, demonstrating that modifying the side-chain structure to alter pharmacokinetics is a viable strategy for expanding therapeutic utility beyond dermatology.

### 13.5. Eldecalcitol (ED-71)

Eldecalcitol (1α,25-dihydroxy-2β-(3-hydroxypropoxy) vitamin D3) represents a unique class of VDR ligands developed for osteoporosis. In contrast to other analogs, eldecalcitol possesses a higher affinity for DBP than the natural ligand 1,25(OH)_2_D_3_, leading to a prolonged plasma half-life and stable distribution to bone tissues [118]. Mechanistically, eldecalcitol is a potent inhibitor of bone resorption. Uniquely, it minimizes the suppression of bone formation compared to classic anti-resorptives, leading to a net increase in bone mineral density [119]. Large-scale clinical trials have demonstrated that eldecalcitol is superior to alfacalcidol in preventing osteoporotic fractures [88,89]. Its efficacy is particularly notable in countries like Japan, where low dietary calcium intake may enhance the drug’s benefits [37].

### 13.6. Emerging Non-Secosteroidal Ligands

Recent medicinal chemistry efforts have shifted toward the development of non-secosteroidal VDR ligands, representing a radical departure from traditional vitamin D backbone design [100,120]. While classic analogs retain the secosteroid scaffold (the broken B-ring structure), they remain inherently susceptible to rapid metabolic degradation by CYP24A1. To overcome this, non-secosteroidal compounds are built upon novel chemical skeletons such as biphenyl, pyrrole, or naphthalene derivatives that spatially mimic the key hydroxyl groups required for VDR binding but lack the labile triene system [105,120,121]. A prime example is LG190178, identified through high-throughput screening. Crystal structure analysis reveals that although it lacks the CD-ring system of calcitriol, it stabilizes the VDR ligand-binding domain in an active conformation, effectively recruiting co-activators [101,111]. Crucially, these ligands induce a unique receptor conformation distinct from that of 1,25(OH)_2_D_3_. This conformational difference promotes “functional selectivity” (or biased signaling), allowing the differential recruitment of co-regulators [101,112]. This mechanism offers the potential to dissociate therapeutic anti-inflammatory or anti-proliferative actions from undesirable calcemic toxicity more effectively than is possible with secosteroidal analogs [101,113]. Furthermore, these next-generation ligands exhibit superior physicochemical properties, including enhanced metabolic stability and oral bioavailability. Although currently in the preclinical stage, they hold significant promise for expanding the therapeutic repertoire for chronic disorders such as autoimmune diseases, metabolic syndrome, and cancer, where long-term VDR activation without hypercalcemia is required [114,115].

## 14. VDR Antagonists and Clinical Application

**Figure 5:**
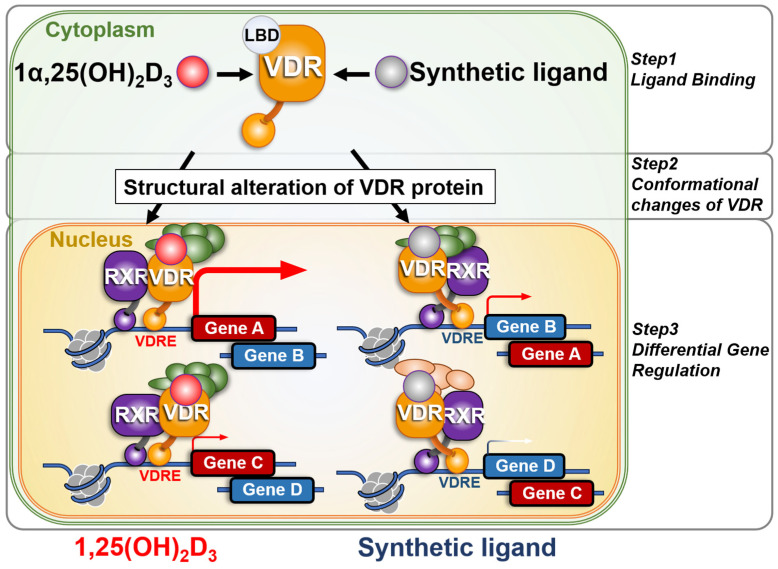
**Structural Alterations of VDR Induced by Synthetic Ligands**. Ligand binding induces conformational changes in the C-terminal region of the vitamin D receptor (VDR) ligand-binding domain, enabling interaction with transcriptional co-regulators [48,49,50]. Synthetic VDR ligands may induce alternative VDR conformations compared with 1,25(OH)_2_D_3_, potentially resulting in tissue- or cell-specific gene expression profiles due to structural differences in the VDR/retinoid X receptor heterodimer complex.

**Figure 6:**
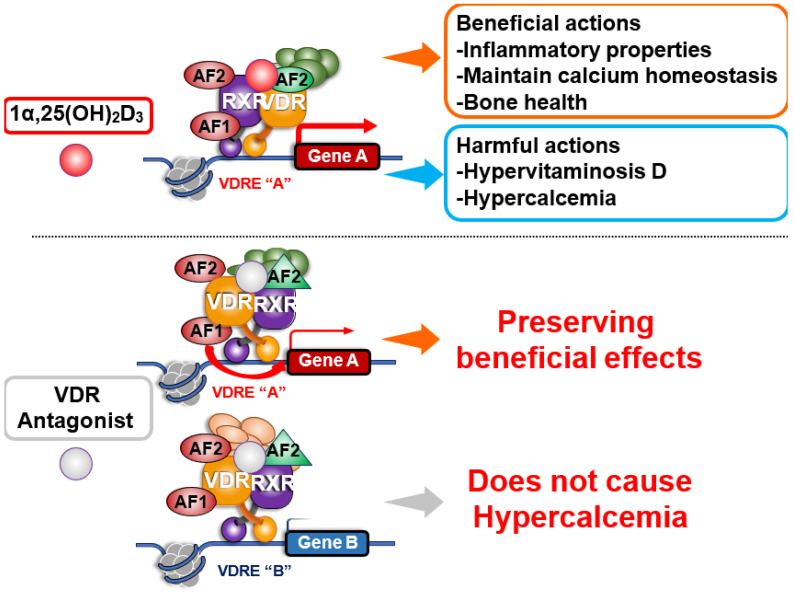
**Beneficial Effects of a VDR Antagonist (a Perspective)**. The calcemic activity of vitamin D can limit its clinical use at high doses. A vitamin D receptor antagonist that selectively blocks calcemic effects while preserving the beneficial actions of vitamin D, such as anti-inflammatory properties, could offer therapeutic advantages, particularly in conditions such as hypervitaminosis D or hyperparathyroidism.

Because the calcemic effects of vitamin D are linked to the transcriptional activity of VDR, any VDR agonist is likely to induce hypercalcemia [10,78], particularly since synthetic ligands are typically selected based on their transactivation potential. As a result, the clinical application of such compounds is limited by the risk of calcium overload [10,27]. In this context, VDR antagonists offer a promising alternative, potentially retaining the beneficial actions of vitamin D while minimizing its calcemic activity (Figure 5), owing to the tissue- or cell type-specific functions of the VDR/RXR heterodimer [37]. This situation is reminiscent of selective estrogen receptor modulators (SERMs), such as raloxifene, which exhibit tissue-specific agonist or antagonist activity [133,134,135]. X-ray crystallographic analyses have revealed that SERMs induce conformational changes in the LBD of estrogen receptor α (ERα), thereby altering its interaction with co-regulators and leading to distinct gene expression patterns in different tissues [136]. Similarly, we found that DLAM-2b, a partial VDR agonist/antagonist, induced a gene expression profile in human cell lines that differed from that induced by 1,25(OH)_2_D_3_ [74,137]. Based on these findings and the SERM [134,136] and selective androgen receptor modulator (SARM) [138] models, it is likely that DLAM-2b induces a different conformation in the VDR than that triggered by 1,25(OH)_2_D_3_ [89]. This idea is further supported by studies of the crystal structure and hydrogen–deuterium exchange in zebrafish VDR LBD [113,125,139,140], which demonstrated that the VDR antagonist ZK16828 fails to induce the active conformation required for co-activator recruitment (Figure 6).

Recently, a group led by Laverny and Metzger characterized a VDR antagonist (ZK168281) referred to as ZK [114]. This compound was selected from over 300 synthetic vitamin D analogs using a variety of medicinal chemistry screening methods. Although ZK had previously been shown to function as a VDR antagonist in rat intestinal cells and human fibroblasts [140,141,142], their study confirmed its antagonistic activity in vivo. In mice administered high doses of 1,25(OH)_2_D_3_, ZK reversed both hypercalcemia and the upregulation of VDR target genes [116].

Mechanistically, ZK exhibits a unique mode of action; it retains VDR in the cytosol by interacting with cellular protein WBP4 [114,115]. This interaction is thought to result from ZK-induced conformational changes in the VDR structure. Additionally, ZK effectively inhibited the expression of 1,25(OH)_2_D_3_-responsive genes in fibroblasts derived from a patient with a CYP24A1 mutation—a condition that impairs inactivation of active vitamin D. These findings suggest that ZK may serve as a potential treatment for vitamin D toxicity (hypervitaminosis D) and hypercalcemia caused by elevated concentrations of 1,25(OH)_2_D_3_ [114].

Vitamin D toxicity can occur in patients with excessive expression of CYP27B1 or impaired CYP24A1 activity [143,144]. These enzymatic mechanisms for regulating 1,25(OH)_2_D_3_ levels have been validated in rodent models disrupted CYP27B1 and CYP24A1 genes [46,145,146]. Overexpression of CYP27B1 is observed in granulomatous diseases such as sarcoidosis and tuberculosis, as well as in certain lymphomas [147]. CYP24A1 dysfunction results from inherited mutations [148]. Since hypervitaminosis D can lead to hypercalcemia, nephrocalcinosis, and hypoparathyroidism, VDR antagonists may be useful in managing these conditions [149]. They may also help lower PTH concentrations in patients with hyperparathyroidism. However, it is noteworthy that VDR antagonists may instead diminish the beneficial activity of natural vitamin D in immune responses.

Moreover, it is worth investigating whether the recently identified SCAP-mediated signaling pathway contributes to anti-inflammatory effects during vitamin D activity (Figure 6). The ZK study suggests additional therapeutic possibilities. As optimal vitamin D is associated with protection against various lifestyle-related diseases through anti-inflammatory mechanisms [2,4,150], generation of a synthetic VDR ligand capable of exerting anti-inflammatory effects without inducing calcemia would be highly desirable. Although the precise molecular basis of the anti-inflammatory effects of vitamin D remain unclear, several studies suggest that ligand-bound VDRs suppress inflammatory signaling pathways [151,152,153]. It is plausible that certain VDR conformations selectively support anti-inflammatory activity without triggering calcium-related effects [154,155].

## 15. Conclusions

Vitamin D and its receptor (VDR) play multifaceted roles in maintaining calcium homeostasis, bone metabolism, and immune regulation. The elucidation of both genomic and non-genomic VDR signaling pathways has provided a robust foundation for designing synthetic vitamin D analogs. While classical VDR agonists have historically struggled to demonstrate efficacy in cancer therapy due to dose-limiting calcemic toxicity, the development of selective VDR modulators and antagonists offers a promising alternative. Compounds such as eldecalcitol and ZK168281 exemplify how structure-based design can achieve tissue-selective effects, minimizing adverse calcium-related outcomes while preserving beneficial properties. Furthermore, the discovery of SCAP-mediated signaling and the regulatory roles of non-coding RNAs has expanded the scope of vitamin D biology beyond skeletal physiology. Crucially, however, therapeutic outcomes are not determined by ligand structure alone; host genetic factors, such as polymorphisms in metabolic enzymes like CYP24A1, and epigenetic variations can impede the proper metabolism or signaling of these analogs. Consequently, future research must integrate structural biology with pharmacogenomics to fully realize the potential of next-generation VDR-targeting agents for treating inflammatory, metabolic, and malignant diseases.

## Data Availability

No new data were created or analyzed in this study. Data sharing is not applicable to this article.

## References

[1] Norman A.W. (2008). From vitamin D to hormone D: Fundamentals of the vitamin D endocrine system essential for good health. Am. J. Clin. Nutr..

[2] Holick M.F., Chen T.C. (2008). Vitamin D deficiency: A worldwide problem with health consequences. Am. J. Clin. Nutr..

[3] Morris H.A., Anderson P.H. (2010). Autocrine and paracrine actions of vitamin D. Clin. Biochem. Rev..

[4] Bouillon R., Carmeliet G., Verlinden L., van Etten E., Verstuyf A., Luderer H.F., Lieben L., Mathieu C., Demay M. (2008). Vitamin D and human health: Lessons from vitamin D receptor null mice. Endocr. Rev..

[5] Park P.G., Lim S.H., Lee H., Ahn Y.H., Cheong H.I., Kang H.G. (2021). Genotype and phenotype analysis in X-linked hypophosphatemia. Front. Pediatr..

[6] Jonsson K.B., Zahradnik R., Larsson T., White K.E., Sugimoto T., Imanishi Y., Yamamoto T., Hampson G., Koshiyama H., Ljunggren Ö. (2003). Fibroblast growth factor 23 in oncogenic osteomalacia and X-linked hypophosphatemia. N. Engl. J. Med..

[7] Rajakumar K. (2003). Vitamin D, cod-liver oil, sunlight, and rickets: A historical perspective. Pediatrics.

[8] Wolf G. (2004). The discovery of vitamin D: The contribution of Adolf windaus. J. Nutr..

[9] Bouillon R., Manousaki D., Rosen C., Trajanoska K., Rivadeneira F., Richards J.B. (2022). The health effects of vitamin D supplementation: Evidence from human studies. Nat. Rev. Endocrinol..

[10] Leyssens C., Verlinden L., Verstuyf A. (2014). The future of vitamin D analogs. Front. Physiol..

[11] Zhang Y., Fang F., Tang J., Jia L., Feng Y., Xu P., Faramand A. (2019). Association between vitamin D supplementation and mortality: Systematic review and meta-analysis. BMJ.

[12] Malloy P.J., Xu R., Peng L., Clark P., Feldman D. (2002). A novel mutation in helix 12 of the vitamin D receptor impairs coactivator interaction and causes hereditary 1,25-dihydroxyvitamin D-resistant rickets without alopecia. Mol. Endocrinol..

[13] Asano L., Watanabe M., Ryoden Y., Usuda K., Yamaguchi T., Khambu B., Takashima M., Sato S.-I., Sakai J., Nagasawa K. (2017). Vitamin D metabolite, 25-hydroxyvitamin D, regulates lipid metabolism by inducing degradation of SREBP/SCAP. Cell Chem. Biol..

[14] Holick M.F., Binkley N.C., Bischoff-Ferrari H.A., Gordon C.M., Hanley D.A., Heaney R.P., Murad M.H., Weaver C.M. (2011). Evaluation, treatment, and prevention of vitamin D deficiency: An Endocrine Society clinical practice guideline. J. Clin. Endocrinol. Metab..

[15] Jodar E., Campusano C., de Jongh R.T., Holick M.F. (2023). Calcifediol: A review of its pharmacological characteristics and clinical use in correcting vitamin D deficiency. Eur. J. Nutr..

[16] Miyamoto H., Kawakami D., Hanafusa N., Nakanishi T., Miyasaka M., Furutani Y., Ikeda Y., Ito K., Kato T., Yokoyama K. (2023). Determination of a Serum 25-Hydroxyvitamin D Reference Ranges in Japanese Adults Using Fully Automated Liquid Chromatography–Tandem Mass Spectrometry. J. Nutr..

[17] MacLaughlin J., Holick M.F. (1985). Aging decreases the capacity of human skin to produce vitamin D_3_. J. Clin. Investig..

[18] Manson J.E., Cook N.R., Lee I.-M., Christen W., Bassuk S.S., Mora S., Gibson H., Gordon D., Copeland T., D’Agostino D. (2019). Vitamin D Supplements and Prevention of Cancer and Cardiovascular Disease. N. Engl. J. Med..

[19] Greten F.R., Grivennikov S.I. (2019). Inflammation and Cancer: Triggers, Mechanisms, and Consequences. Immunity.

[20] Furman D., Campisi J., Verdin E., Carrera-Bastos P., Targ S., Franceschi C., Ferrucci L., Gilroy D.W., Fasano A., Miller G.W. (2019). Chronic inflammation in the etiology of disease across the life span. Nat. Med..

[21] Yin K., Agrawal D.K. (2014). Vitamin D and inflammatory diseases. J. Inflamm. Res..

[22] Acen E.L., Biraro I.A., Worodria W., Joloba M.L., Nkeeto B., Musaazi J., Kateete D.P. (2021). Impact of vitamin D status and cathelicidin antimicrobial peptide on adults with active pulmonary TB globally: A systematic review and meta-analysis. PLoS ONE.

[23] Liu P.T., Stenger S., Li H., Wenzel L., Tan B.H., Krutzik S.R., Ochoa M.T., Schauber J., Wu K., Meinken C. (2006). Toll-like receptor triggering of a vitamin D-mediated human antimicrobial response. Science.

[24] Callejo M., Morales-Cano D., Olivencia M.A., Mondejar-Parreño G., Barreira B., Tura-Ceide O., Martínez V.G., Serrano-Navarro A., Moreno L., Morrell N. (2024). Vitamin D receptor and its antiproliferative effect in human pulmonary arterial hypertension. Sci. Rep..

[25] Seraphin G., Rieger S., Hewison M., Capobianco E., Lisse T.S. (2023). The impact of vitamin D on cancer: A mini review. J. Steroid Biochem. Mol. Biol..

[26] Wimalawansa S.J. (2025). Vitamin D’s impact on cancer incidence and mortality: A systematic review. Nutrients.

[27] Bouillon R., Marcocci C., Carmeliet G., Bikle D., White J.H., Dawson-Hughes B., Lips P., Munns C.F., Lazaretti-Castro M., Giustina A. (2019). Skeletal and extraskeletal actions of vitamin D: Current evidence and outstanding questions. Endocr. Rev..

[28] Verlinden L., Carmeliet G. (2021). Integrated view on the role of vitamin D actions on bone and growth plate homeostasis. JBMR Plus.

[29] Suda T., Takahashi N., Udagawa N., Jimi E., Gillespie M.T., Martin T.J. (1999). Modulation of osteoclast differentiation and function by the new members of the tumor necrosis factor receptor and ligand families. Endocr. Rev..

[30] Khan A.A., Hanley D.A., Rizzoli R., Bollerslev J., Young J.E.M., Rejnmark L., Thakker R., D’amour P., Paul T., Van Uum S. (2017). Primary hyperparathyroidism: Review and recommendations on evaluation, diagnosis, and management. A Canadian and international consensus. Osteoporos. Int..

[31] Jones G. (2008). Pharmacokinetics of vitamin D toxicity. Am. J. Clin. Nutr..

[32] Takeyama K.-I., Kitanaka S., Sato T., Kobori M., Yanagisawa J., Kato S. (1997). 25-hydroxyvitamin D_3_ 1α-hydroxylase and vitamin D synthesis. Science.

[33] Jones G., Prosser D.E., Kaufmann M. (2014). Cytochrome P450-mediated metabolism of vitamin D. J. Lipid Res..

[34] Brenza H.L., Kimmel-Jehan C., Jehan F., Shinki T., Wakino S., Anazawa H., Suda T., DeLuca H.F. (1998). Parathyroid hormone activation of the 25-hydroxyvitamin D_3_-1α-hydroxylase gene promoter. Proc. Natl. Acad. Sci. USA.

[35] Kanemoto Y., Nishimura K., Hayakawa A., Sawada T., Amano R., Mori J., Kurokawa T., Murakami Y., Kato S. (2022). A long non-coding RNA as a direct vitamin D target transcribed from the antisense strand of the human HSD17B2 locus. Biosci. Rep..

[36] Zierold C., Darwish H.M., DeLuca H.F. (1995). Two vitamin D response elements function in the rat 1,25-dihydroxyvitamin D 24-hydroxylase promoter. J. Biol. Chem..

[37] Kanemoto Y., Iwaki M., Sawada T., Nojiri K., Kurokawa T., Tsutsumi R., Nagasawa K., Kato S. (2023). Advances in the administration of vitamin D analogues to support bone health and treat chronic diseases. J. Bone Metab..

[38] Ao T., Kikuta J., Ishii M. (2021). The effects of vitamin D on immune system and inflammatory diseases. Biomolecules.

[39] Stachowicz-Suhs M., Łabędź N., Milczarek M., Kłopotowska D., Filip-Psurska B., Maciejczyk A., Matkowski R., Wietrzyk J. (2024). Vitamin D_3_ reduces the expression of M1 and M2 macrophage markers in breast cancer patients. Sci. Rep..

[40] Yuk J.-M., Shin D.-M., Lee H.-M., Yang C.-S., Jin H.S., Kim K.-K., Lee Z.-W., Lee S.-H., Kim J.-M., Jo E.-K. (2009). Vitamin D_3_ induces autophagy in human monocytes/macrophages via cathelicidin. Cell Host Microbe.

[41] Kitanaka S., Takeyama K., Murayama A., Sato T., Okumura K., Nogami M., Hasegawa Y., Niimi H., Yanagisawa J., Tanaka T. (1998). Inactivating mutations in the 25-hydroxyvitamin D_3_ 1α-hydroxylase gene in patients with pseudovitamin D-deficiency rickets. N. Engl. J. Med..

[42] Hughes M.R., Malloy P.J., Kieback D.G., Kesterson R.A., Pike J.W., Feldman D., O’Malley B.W. (1988). Point mutations in the human vitamin D receptor gene associated with hypocalcemic rickets. Science.

[43] Fraser D.R., Kodicek E. (1970). Unique human syndrome hereditary resistance vitamin D. Lancet.

[44] Miller J., Gallo R.L. (2010). Vitamin D: New era dermatologic medicine. Semin. Cutan. Med. Surg..

[45] Yoshizawa T., Handa Y., Uematsu Y., Takeda S., Sekine K., Yoshihara Y., Kawakami T., Arioka K., Sato H., Uchiyama Y. (1997). Mice lacking the vitamin D receptor exhibit impaired bone formation, uterine hypoplasia and growth retardation after weaning. Nat. Genet..

[46] Panda D.K., Miao D., Tremblay M.L., Sirois J., Farookhi R., Hendy G.N., Goltzman D. (2001). Targeted ablation of the 25-hydroxyvitamin D 1α-hydroxylase enzyme: Evidence for skeletal, reproductive, and immune dysfunction. Proc. Natl. Acad. Sci. USA.

[47] Li Y.C., Pirro A.E., Amling M., Delling G., Baron R., Bronson R., Demay M.B. (1997). Targeted ablation of the vitamin D receptor: An animal model of vitamin D-dependent rickets type II with alopecia. Proc. Natl. Acad. Sci. USA.

[48] Glass C.K., Rosenfeld M.G. (2000). The coregulator exchange in transcriptional functions of nuclear receptors. Genes Dev..

[49] Kato S., Yokoyama A., Fujiki R. (2011). Nuclear receptor coregulators merge transcriptional coregulation with epigenetic regulation. Trends Biochem. Sci..

[50] Meyer M.B., Goetsch P.D., Pike J.W. (2010). A downstream intergenic cluster of regulatory enhancers contributes to the induction of CYP24A1 expression by 1α,25-dihydroxyvitamin D_3_. J. Biol. Chem..

[51] Malloy P.J., Pike J.W., Feldman D. (1999). The vitamin D receptor and the syndrome of hereditary 1,25-dihydroxyvitamin D-resistant rickets. Endocr. Rev..

[52] Kise S., Morita S., Sakaki T., Kimura H., Kinuya S., Yasuda K. (2025). Ligand-independent vitamin D receptor actions essential for keratinocyte homeostasis in the skin. Int. J. Mol. Sci..

[53] Joko Y., Yamamoto Y., Kato S., Takemoto T., Abe M., Matsumoto T., Fukumoto S., Sawatsubashi S. (2023). VDR is an essential regulator of hair follicle regression through the progression of cell death. Life Sci. Alliance.

[54] Xue Y., Fleet J.C. (2009). Intestinal vitamin D receptor is required for normal calcium and bone metabolism in mice. Gastroenterology.

[55] Kristjansson K., Rut R., Hewison M., Oriordan J.L.H., Hughes M.R. (1993). Two mutations in the hormone binding domain of the vitamin D receptor cause tissue resistance to 1,25 dihydroxyvitamin D_3_. J. Clin. Investig..

[56] Eelen G., Verlinden L., Rochel N., Claessens F., De Clercq P., Vandewalle M., Tocchini-Valentini G., Moras D., Bouillon R., Verstuyf A. (2005). Superagonistic action of 14-epi-analogs of 1,25-dihydroxyvitamin D explained by vitamin D receptor-coactivator interaction. Mol. Pharmacol..

[57] Nishikawa M., Yasuda K., Takamatsu M., Abe K., Okamoto K., Horibe K., Mano H., Nakagawa K., Tsugawa N., Hirota Y. (2020). Generation of novel genetically modified rats to reveal the molecular mechanisms of vitamin D actions. Sci. Rep..

[58] Masuyama R., Nakaya Y., Tanaka S., Tsurukami H., Nakamura T., Watanabe S., Yoshizawa T., Kato S., Suzuki K. (2001). Dietary phosphorus restriction reverses the impaired bone mineralization in vitamin D receptor knockout mice. Endocrinology.

[59] Mori T., Horibe K., Koide M., Uehara S., Yamamoto Y., Kato S., Yasuda H., Takahashi N., Udagawa N., Nakamichi Y. (2020). The vitamin D receptor in osteoblast-lineage cells is essential for the proresorptive activity of 1α,25(OH)_2_D_3_ In Vivo. Endocrinology.

[60] Lieben L., Carmeliet G. (2013). Vitamin D signaling in osteocytes: Effects on bone and mineral homeostasis. Bone.

[61] Evans R.M., Mangelsdorf D.J. (2014). Nuclear receptors, RXR, and the Big Bang. Cell.

[62] Bikle D.D. (2021). Ligand-independent actions of the vitamin D receptor: More questions than answers. JBMR Plus.

[63] Skorija K., Cox M., Sisk J.M., Dowd D.R., MacDonald P.N., Thompson C.C., Demay M.B. (2005). Ligand-independent actions of the vitamin D receptor maintain hair follicle homeostasis. Mol. Endocrinol..

[64] Benavides F., Oberyszyn T.M., VanBuskirk A.M., Reeve V.E., Kusewitt D.F. (2009). The hairless mouse in skin research. J. Dermatol. Sci..

[65] Kim J.Y., Son Y.L., Lee Y.C. (2009). Involvement of SMRT corepressor in transcriptional repression by the vitamin D receptor. Mol. Endocrinol..

[66] Raisz L.G., Trummel C.L., Holick M.F., DeLuca H.F. (1972). 1,25-dihydroxycholecalciferol: A potent stimulator of bone resorption in tissue culture. Science.

[67] Takeda S., Yoshizawa T., Nagai Y. (1999). Stimulation osteoclast formation 1,25-dihydroxyvitamin D_3_ bone marrow cultures from vitamin D receptor knockout mice. Endocrinology.

[68] van de Peppel J., van Leeuwen J.P.T.M. (2014). Vitamin D and gene networks in human osteoblasts. Front. Physiol..

[69] Nakamichi Y., Udagawa N., Horibe K., Mizoguchi T., Yamamoto Y., Nakamura T., Hosoya A., Kato S., Suda T., Takahashi N. (2017). VDR in osteoblast-lineage cells primarily mediates vitamin D treatment-induced increase in bone mass by suppressing bone resorption. J. Bone Miner. Res..

[70] Yamamoto Y., Yoshizawa T., Fukuda T., Shirode-Fukuda Y., Yu T., Sekine K., Sato T., Kawano H., Aihara K.-I., Nakamichi Y. (2013). Vitamin D receptor in osteoblasts is a negative regulator of bone mass control. Endocrinology.

[71] Fleet J.C., Reyes-Fernandez P. (2020). Intestinal responses to 1,25 dihydroxyvitamin D are not improved by higher intestinal VDR levels resulting from intestine-specific transgenic expression of VDR in mice. J. Steroid Biochem. Mol. Biol..

[72] Li Y.C., Bolt M.J., Cao L.P., Sitrin M.D. (2001). Effects of vitamin D receptor inactivation on the expression of calbindins and calcium metabolism. Am. J. Physiol. Endocrinol. Metab..

[73] Sawatsubashi S., Nishimura K., Mori J., Kouzmenko A., Kato S. (2019). The Function of the Vitamin D Receptor and a Possible Role of Enhancer RNA in Epigenomic Regulation of Target Genes: Implications for Bone Metabolism. J. Bone Metab..

[74] Hii C.S., Ferrante A. (2016). The non-genomic actions of vitamin D. Nutrients.

[75] Nemere I., Garbi N., Hämmerling G.J., Khanal R.C. (2010). Intestinal cell calcium uptake and the targeted knockout of the 1,25D_3_-MARRS (membrane-associated, rapid response steroid-binding) receptor/PDIA3/Erp57. J. Biol. Chem..

[76] Żmijewski M.A. (2022). Nongenomic activities of vitamin D. Nutrients.

[77] Kawagoe F., Mendoza A., Hayata Y., Asano L., Kotake K., Mototani S., Kawamura S., Kurosaki S., Akagi Y., Takemoto Y. (2021). Discovery of a vitamin D receptor-silent vitamin D derivative that impairs sterol regulatory element-binding protein in vivo. J. Med. Chem..

[78] Christakos S., Dhawan P., Verstuyf A., Verlinden L., Carmeliet G. (2016). Vitamin D: Metabolism, Molecular Mechanism of Action, and Pleiotropic Effects. Physiol. Rev..

[79] Umesono K., Murakami K.K., Thompson C.C., Evans R.M. (1991). Direct repeats as selective response elements for the thyroid hormone, retinoic acid, and vitamin D_3_ receptors. Cell.

[80] Kato S. (2000). The function of vitamin D receptor in vitamin D action. J. Biochem..

[81] Haussler M.R., Whitfield G.K., Haussler C.A., Hsieh J.C., Thompson P.D., Selznick S.H., Dominguez C.E., Jurutka P.W. (1998). The nuclear vitamin D receptor: Biological and molecular regulatory properties revealed. J. Bone Miner. Res..

[82] Li W., Notani D., Rosenfeld M.G. (2016). Enhancers as non-coding RNA transcription units: Recent insights and future perspectives. Nat. Rev. Genet..

[83] Nair S.J., Yang L., Meluzzi D., Oh S., Yang F., Friedman M.J., Wang S., Suter T., Alshareedah I., Gamliel A. (2019). Phase separation of ligand-activated enhancers licenses cooperative chromosomal enhancer assembly. Nat. Struct. Mol. Biol..

[84] Boija A., Klein I.A., Sabari B.R., Dall’Agnese A., Coffey E.L., Zamudio A.V., Li C.H., Shrinivas K., Manteiga J.C., Hannett N.M. (2018). Transcription factors activate genes through the phase-separation capacity of their activation domains. Cell.

[85] Tagami T., Lutz W.H., Kumar R., Jameson J.L. (1998). The interaction of the vitamin D receptor with nuclear receptor corepressors and coactivators. Biochem. Biophys. Res. Commun..

[86] Allen B.L., Taatjes D.J. (2015). The Mediator complex: A central integrator of transcription. Nat. Rev. Mol. Cell Biol..

[87] Oñate S.A., Tsai S.Y., Tsai M.J., O’Malley B.W. (1995). Sequence and characterization of a coactivator for the steroid hormone receptor superfamily. Science.

[88] Chen T., Dent S.Y.R. (2014). Chromatin modifiers and remodellers: Regulators of cellular differentiation. Nat. Rev. Genet..

[89] Iwaki M., Kanemoto Y., Sawada T., Nojiri K., Kurokawa T., Tsutsumi R., Nagasawa K., Kato S. (2023). Differential gene regulation by a synthetic vitamin D receptor ligand and active vitamin D in human cells. PLoS ONE.

[90] Wei Z., Yoshihara E., He N., Hah N., Fan W., Pinto A.F.M., Huddy T., Wang Y., Ross B., Estepa G. (2018). Vitamin D Switches BAF Complexes to Protect β Cells. Cell.

[91] Matsumoto T., Yamamoto K., Takeuchi T., Tanaka Y., Tanaka S., Nakano T., Ito M., Tomomitsu T., Hirakawa A., Soen S. (2020). Eldecalcitol is superior to alfacalcidol in maintaining bone mineral density in glucocorticoid-induced osteoporosis patients (e-GLORIA). J. Bone Miner. Metab..

[92] Matsumoto T., Ito M., Hayashi Y., Hirota T., Tanigawara Y., Sone T., Fukunaga M., Shiraki M., Nakamura T. (2011). A new active vitamin D_3_ analog, eldecalcitol, prevents the risk of osteoporotic fractures—A randomized, active comparator, double-blind study. Bone.

[93] Brown A.J., Slatopolsky E. (2007). Drug insight: Vitamin D analogs in the treatment of secondary hyperparathyroidism in patients with chronic kidney disease. Nat. Clin. Pract. Endocrinol. Metab..

[94] Bouillon R., Okamura W.H., Norman A.W. (1995). Structure-function relationships in the vitamin D endocrine system. Endocr. Rev..

[95] Prudencio J., Akutsu N., Benlimame N., Wang T., Bastien Y., Lin R., Black M.J., Alaoui-Jamali M.A., White J.H. (2001). Action of low calcemic 1α,25-dihydroxyvitamin D_3_ analogue EB1089 in head and neck squamous cell carcinoma. J. Natl. Cancer Inst..

[96] Hansen C.M., Hamberg K.J., Binderup E., Binderup L. (2000). Seocalcitol (EB 1089): A vitamin D analogue of anti-cancer potential. Background, design, synthesis, pre-clinical and clinical evaluation. Curr. Pharm. Des..

[97] Kragballe K., Beck H.I., Søgaard H. (1988). Improvement of psoriasis by a topical vitamin D_3_ analogue (MC 903) in a double-blind study. Br. J. Dermatol..

[98] Kragballe K. (1996). Wild-type mutant vitamin D receptors psoriasis. J. Investig. Dermatol. Symp. Proc..

[99] Tocchini-Valentini G., Rochel N., Wurtz J.-M., Moras D. (2004). Crystal structures of the vitamin D nuclear receptor liganded with the vitamin D side chain analogues calcipotriol and seocalcitol, receptor agonists of clinical importance. Insights into a structural basis for the switching of calcipotriol to a receptor antagonist by further side chain modification. J. Med. Chem..

[100] Franchi M., Gunnarsson J., Gonzales-Parra E., Ferreira A., Ström O., Corrao G. (2023). Paricalcitol and extended-release calcifediol for treatment of secondary hyperparathyroidism in non-dialysis chronic kidney disease: Results from a network meta-analysis. J. Clin. Endocrinol. Metab..

[101] Barker J.N., Ashton R.E., Marks R., Harris R.I., Berth-Jones J. (1999). Topical maxacalcitol for the treatment of psoriasis vulgaris: A placebo-controlled, double-blind, dose-finding study with active comparator. Br. J. Dermatol..

[102] Pepper C., Thomas A., Hoy T., Milligan D., Bentley P., Fegan C. (2003). The vitamin D_3_ analog EB1089 induces apoptosis via a p53-independent mechanism involving p38 MAP kinase activation and suppression of ERK activity in B-cell chronic lymphocytic leukemia cells in vitro. Blood.

[103] Patel R., Nandini Kharkwal H., Saha M., Sankaranarayanan M., Sharma S., Chander S. (2025). Recent advancements towards the use of vitamin D isoforms and the development of their synthetic analogues as new therapeutics. Biomedicines.

[104] Boehm M.F., Fitzgerald P., Zou A., Elgort M.G., Bischoff E.D., Mere L., Mais D.E., Bissonnette R.P., Heyman R.A., Nadzan A.M. (1999). Novel nonsecosteroidal vitamin D mimics exert VDR-modulating activities with less calcium mobilization than 1,25-dihydroxyvitamin D_3_. Chem. Biol..

[105] Sundaram S., Sea A., Feldman S., Strawbridge R., Hoopes P.J., Demidenko E., Binderup L., Gewirtz A.D. (2003). The combination of a potent vitamin D_3_ analog, EB 1089, with ionizing radiation reduces tumor growth and induces apoptosis of MCF-7 breast tumor xenografts in nude mice. Clin. Cancer Res..

[106] Dalhoff K., Dancey J., Astrup L., Skovsgaard T., Hamberg K.J., Lofts F.J., Rosmorduc O., Erlinger S., Hansen J.B., Steward W.P. (2003). A phase II study of the vitamin D analogue Seocalcitol in patients with inoperable hepatocellular carcinoma. Br. J. Cancer.

[107] Lebwohl M.G. (1995). The evolution of vitamin D analogues for the treatment of psoriasis. Arch. Dermatol..

[108] Mortensen L., Kragballe K., Wegmann E., Schifter S., Risteli J., Charles P. (1993). Treatment of psoriasis vulgaris with topical calcipotriol has no short-term effect on calcium or bone metabolism. A randomized, double-blind, placebo-controlled study. Acta Derm. Venereol..

[109] Geng X., Shi E., Wang S., Song Y. (2020). A comparative analysis of the efficacy and safety of paricalcitol versus other vitamin D receptor activators in patients undergoing hemodialysis: A systematic review and meta-analysis of 15 randomized controlled trials. PLoS ONE.

[110] Martin K.J., González E.A., Gellens M., Hamm L.L., Abboud H., Lindberg J. (1998). 19-Nor-1-α-25-dihydroxyvitamin D2 (Paricalcitol) safely and effectively reduces the levels of intact parathyroid hormone in patients on hemodialysis. J. Am. Soc. Nephrol..

[111] Tada H., Shimizu T., Nagaoka I., Takada H. (2016). Vitamin D_3_ analog maxacalcitol (OCT) induces hCAP-18/LL-37 production in human oral epithelial cells. Biomed. Res..

[112] Posner G.H., Helvig C., Cuerrier D., Collop D., Kharebov A., Ryder K., Epps T., Petkovich M. (2010). Vitamin D analogues targeting CYP24 in chronic kidney disease. J. Steroid Biochem. Mol. Biol..

[113] Belorusova A.Y., Chalhoub S., Rovito D., Rochel N. (2020). Structural analysis of VDR complex with ZK168281 antagonist. J. Med. Chem..

[114] Rovito D., Belorusova A.Y., Chalhoub S., Rerra A.-I., Guiot E., Molin A., Linglart A., Rochel N., Laverny G., Metzger D. (2020). Cytosolic sequestration of the vitamin D receptor as a therapeutic option for vitamin D-induced hypercalcemia. Nat. Commun..

[115] Sudol M. (1996). The WW module competes with the SH3 domain?. Trends Biochem. Sci..

[116] Bury Y., Steinmeyer A., Carlberg C. (2000). Structure activity relationship of carboxylic ester antagonists of the vitamin D_3_ receptor. Mol. Pharmacol..

[117] Kashiwagi H., Ono Y., Ohta M., Itoh S., Ichikawa F., Harada S., Takeda S., Sekiguchi N., Ishigai M., Takahashi T. (2013). A series of nonsecosteroidal vitamin D receptor agonists for osteoporosis therapy. Bioorg. Med. Chem..

[118] Khedkar S.A., Samad M.A., Choudhury S., Lee J.Y., Zhang D., Thadhani R.I., Karumanchi S.A., Rigby A.C., Kang P.M. (2017). Identification of novel non-secosteroidal vitamin D receptor agonists with potent cardioprotective effects and devoid of hypercalcemia. Sci. Rep..

[119] Maestro M.A., Seoane S. (2022). The centennial collection of VDR ligands: Metabolites, analogs, hybrids and non-secosteroidal ligands. Nutrients.

[120] Kakuda S., Okada K., Eguchi H., Takenouchi K., Hakamata W., Kurihara M., Takimoto-Kamimura M. (2008). Structure of the ligand-binding domain of rat VDR in complex with the nonsecosteroidal vitamin D_3_ analogue YR301. Acta Crystallogr. Sect. F Struct. Biol. Cryst. Commun..

[121] Inaba Y., Yamamoto K., Yoshimoto N., Matsunawa M., Uno S., Yamada S., Makishima M. (2007). Vitamin D_3_ derivatives with adamantane or lactone ring side chains are cell type-selective vitamin D receptor modulators. Mol. Pharmacol..

[122] Moras D., Gronemeyer H. (1998). The nuclear receptor ligand-binding domain: Structure and function. Curr. Opin. Cell Biol..

[123] Yaghmaei S., Roberts C., Ai R., Mizwicki M.T., Chang C.-E.A. (2013). Agonist and antagonist binding to the nuclear vitamin D receptor: Dynamics, mutation effects and functional implications. Silico Pharmacol..

[124] Molnár F. (2014). Structural considerations of vitamin D signaling. Front. Physiol..

[125] Rochel N., Wurtz J.M., Mitschler A., Klaholz B., Moras D. (2000). The crystal structure of the nuclear receptor for vitamin D bound to its natural ligand. Mol. Cell.

[126] Teichert A., Arnold L.A., Otieno S., Oda Y., Augustinaite I., Geistlinger T.R., Kriwacki R.W., Guy R.K., Bikle D.D. (2009). Quantification of the vitamin D receptor-coregulator interaction. Biochemistry.

[127] Sidhu P.S., Nassif N., McCallum M.M., Teske K., Feleke B., Yuan N.Y., Nandhikonda P., Cook J.M., Singh R.K., Bikle D.D. (2014). Development of novel Vitamin D Receptor-Coactivator Inhibitors. ACS Med. Chem. Lett..

[128] Rochel N., Hourai S., Pérez-García X., Rumbo A., Mourino A., Moras D. (2007). Crystal structure of the vitamin D nuclear receptor ligand binding domain in complex with a locked side chain analog of calcitriol. Arch. Biochem. Biophys..

[129] Mizwicki M.T., Keidel D., Bula C.M., Bishop J.E., Zanello L.P., Wurtz J.-M., Moras D., Norman A.W. (2004). Identification of an alternative ligand-binding pocket in the nuclear vitamin D receptor and its functional importance in 1α,25(OH)_2_-vitamin D_3_ signaling. Proc. Natl. Acad. Sci. USA.

[130] Carlberg C., Molnár F. (2006). Detailed molecular understanding of agonistic and antagonistic vitamin D receptor ligands. Curr. Top. Med. Chem..

[131] Colston K.W., Mackay A.G., James S.Y., Binderup L., Chander S., Coombes R.C. (1992). EB1089: A new vitamin D analogue that inhibits the growth of breast cancer cells in vivo and in vitro. Biochem. Pharmacol..

[132] Sprague S.M., Llach F., Amdahl M., Taccetta C., Batlle D. (2003). Paricalcitol versus calcitriol in the treatment of secondary hyperparathyroidism. Kidney Int..

[133] Jordan V.C. (2004). Selective estrogen receptor modulation: Concept and consequences in cancer. Cancer Cell.

[134] McDonnell D.P., Wardell S.E. (2010). The molecular mechanisms underlying the pharmacological actions of ER modulators: Implications for new drug discovery in breast cancer. Curr. Opin. Pharmacol..

[135] Smith C.L., O’Malley B.W. (2004). Coregulator function: A key to understanding tissue specificity of selective receptor modulators. Endocr. Rev..

[136] Brzozowski A.M., Pike A.C., Dauter Z., Hubbard R.E., Bonn T., Engström O., Öhman L., Greene G.L., Gustafsson J.A., Carlquist M. (1997). Molecular basis of agonism and antagonism in the oestrogen receptor. Nature.

[137] Nakabayashi M., Yamada S., Yoshimoto N., Tanaka T., Igarashi M., Ikura T., Ito N., Makishima M., Tokiwa H., DeLuca H.F. (2008). Crystal structures of rat vitamin D receptor bound to adamantyl vitamin D analogs: Structural basis for vitamin D receptor antagonism and partial agonism. J. Med. Chem..

[138] Nadal M., Prekovic S., Gallastegui N., Helsen C., Abella M., Zielinska K., Gay M., Vilaseca M., Taulès M., Houtsmuller A.B. (2017). Structure of the homodimeric androgen receptor ligand-binding domain. Nat. Commun..

[139] Ozono K., Saito M., Miura D., Michigami T., Nakajima S., Ishizuka S. (1999). Analysis of the molecular mechanism for the antagonistic action of a novel 1α,25-dihydroxyvitamin D_3_ analogue toward vitamin D receptor function. J. Biol. Chem..

[140] Peräkylä M., Molnár F., Carlberg C. (2004). A structural basis for the species-specific antagonism of 26,23-lactones on vitamin D signaling. Chem. Biol..

[141] Herdick M., Steinmeyer A., Carlberg C. (2000). Carboxylic ester antagonists of 1α,25-dihydroxyvitamin D_3_ show cell-specific actions. Chem. Biol..

[142] Herdick M., Steinmeyer A., Carlberg C. (2000). Antagonistic action of a 25-carboxylic ester analogue of 1α, 25-dihydroxyvitamin D_3_ is mediated by a lack of ligand-induced vitamin D receptor interaction with coactivators. J. Biol. Chem..

[143] Marcinowska-Suchowierska E., Kupisz-Urbańska M., Łukaszkiewicz J., Płudowski P., Jones G. (2018). Vitamin D toxicity-A clinical perspective. Front. Endocrinol..

[144] Taylor P.N., Davies J.S. (2018). A review of the growing risk of vitamin D toxicity from inappropriate practice. Br. J. Clin. Pharmacol..

[145] Bikle D., Christakos S. (2020). New aspects of vitamin D metabolism and action—Addressing the skin as source and target. Nat. Rev. Endocrinol..

[146] Yasuda K., Nishikawa M., Okamoto K., Horibe K., Mano H., Yamaguchi M., Okon R., Nakagawa K., Tsugawa N., Okano T. (2021). Elucidation of metabolic pathways of 25-hydroxyvitamin D_3_ mediated by CYP24A1 and CYP3A using Cyp24a1 knockout rats generated by CRISPR/Cas9 system. J. Biol. Chem..

[147] Pao V.Y., Chang S., Shoback D.M., Bikle D.D. (2009). Hypercalcemia and overexpression of CYP27B1 in a patient with nephrogenic systemic fibrosis: Clinical vignette and literature review. J. Bone Miner. Res..

[148] Schlingmann K.P., Kaufmann M., Weber S., Irwin A., Goos C., John U., Misselwitz J., Klaus G., Kuwertz-Bröking E., Fehrenbach H. (2011). Mutations in CYP24A1 and idiopathic infantile hypercalcemia. N. Engl. J. Med..

[149] Tebben P.J., Singh R.J., Kumar R. (2016). Vitamin D-mediated hypercalcemia: Mechanisms, diagnosis, and treatment. Endocr. Rev..

[150] Wang H., Chen W., Li D., Yin X., Zhang X., Olsen N., Zheng S.G. (2017). Vitamin D and chronic diseases. Aging Dis..

[151] Ghaseminejad-Raeini A., Ghaderi A., Sharafi A., Nematollahi-Sani B., Moossavi M., Derakhshani A., Sarab G.A. (2023). Immunomodulatory actions of vitamin D in various immune-related disorders: A comprehensive review. Front. Immunol..

[152] Jaroslawska J., Ghosh Dastidar R., Carlberg C. (2024). In vivo vitamin D target genes interconnect key signaling pathways of innate immunity. PLoS ONE.

[153] Chen Y., Zhang J., Ge X., Du J., Deb D.K., Li Y.C. (2013). Vitamin D receptor inhibits nuclear factor κB activation by interacting with IκB kinase β protein. J. Biol. Chem..

[154] Nagpal S., Na S., Rathnachalam R. (2005). Noncalcemic actions of vitamin D receptor ligands. Endocr. Rev..

[155] Lempiäinen H., Molnár F., Macias Gonzalez M., Peräkylä M., Carlberg C. (2005). Antagonist- and inverse agonist-driven interactions of the vitamin D receptor and the constitutive androstane receptor with corepressor protein. Mol. Endocrinol..

